# High Coronary Wall Shear Stress Worsens Plaque Vulnerability: A Systematic Review and Meta-Analysis

**DOI:** 10.1177/0003319721991722

**Published:** 2021-02-04

**Authors:** Artan Bajraktari, Ibadete Bytyçi, Michael Y. Henein

**Affiliations:** 1 Institute of Public Health and Clinical Medicine, Umea University, Sweden; 2 University College, Bardhosh, Kosovo; 3 Clinic of Cardiology, University Clinical Centre of Kosovo, Prishtina, Kosovo; 4 Molecular and Clinic Research Institute, St George University, London, and Brunel University, United Kingdom

**Keywords:** wall shear stress, coronary artery disease, intravascular ultrasound

## Abstract

**Aim:**

The aim of this meta-analysis is to assess the impact of wall shear stress (WSS) severity on arterial plaque vulnerability.

**Methods:**

We systematically searched electronic databases and selected studies which assessed the relationship between WSS measured by intravascular ultrasound and coronary artery plaque features. In 7 studies, a total of 615 patients with 28 276 arterial segments (median follow-up: 7.71 months) were identified. At follow-up, the pooled analysis showed high WSS to be associated with regression of plaque fibrous area, weighted mean difference (WMD) −0.11 (95% CI: −0.20 to −0.02, *P* = .02) and fibrofatty area, WMD −0.09 (95% CI: −0.17 to −0.01, *P* = .02), reduction in plaque total area, WMD −0.09 (95% CI: −0.14 to −0.04, *P* = .007) and increased necrotic core area, and WMD 0.04 (95% CI: 0.01-0.09, *P* = .03) compared with low WSS. Dense calcium deposits remained unchanged in high and low WSS (0.01 vs 0.02 mm^2^; *P* > .05). High WSS resulted in profound remodeling (40% vs 18%, *P* < .05) and with more constructive remodeling than low WSS (78% vs 40%, *P* < .01).

**Conclusions:**

High WSS in coronary arteries is associated with worsening plaque vulnerability and more profound arterial wall remodeling compared with low WSS.

## Introduction

Atherosclerosis is a major health problem worldwide because of its related high morbidity, hospitalization, and mortality.^1^ Despite major advances in the treatment of coronary artery disease (CAD), a large number of patients who are apparently healthy have a cardiovascular (CV) event or die suddenly without prior symptoms. Available diagnostic techniques are not adequate for identifying patients at high risk of developing events. Recognizing the role of arterial vulnerable plaque as a cause for those events has become an important diagnostic target in CV medicine.^2,3^

Wall shear stress (WSS) is the tangential force produced by viscous blood on the endothelium and it plays an important role in the process of atherosclerosis.^4^ Studies have shown that WSS has different effects on plaque burden and composition,^5^ with high WSS associated with increased plaque vulnerability.^6-8^ However, there is no consensus on the relevance of WSS in clinical practice. If evidence is provided regarding the role of WSS in predicting plaque stability, it would promote further research. The aim of this meta-analysis is to assess the impact of WSS on coronary artery plaque features and vulnerability.

## Methods

We followed the guidelines of the Preferred Reporting Items for Systematic Reviews and Meta-Analysis statement^9^ amendment to the Quality of Reporting of Meta-analyses statement.^10^ Due to the nature of the study design (meta-analysis), neither institutional ethics review board approval nor patient informed consent was needed.

### Data Sources

We systematically searched PubMed, Medline, EMBASE, Scopus, Google Scholar, and the Cochrane Central Registry of Controlled Trials and ClinicalTrial.gov, up to September 2019, using the following key words: “Wall shear stress” OR “WSS” OR “High wall shear stress” OR “High WSS” OR “Intermediate wall shear stress” OR “Intermediate WSS” OR “Low wall shear stress” OR “Low WSS” AND “Coronary artery disease” OR “CAD” OR “Ischemic heart disease” OR “IHD” AND “Intracardiac ultrasound” OR (IVUS) AND “Atherosclerotic plaque” OR “Plaque morphology.”

Additional searches for potential trials which included the references of review articles and the abstracts from selected congresses including the scientific sessions of the European Society of Cardiology, the American Heart Association, American College of Cardiology (ACC), and European Association of Cardiovascular Imaging were also undertaken. The wild card term “*” was used to increase the sensitivity of the search strategy. The literature search was limited to studies in humans and articles published in English. Two reviewers (A.B. and I.B.) independently evaluated each article. No filters were applied. Disagreements were resolved by discussion with a third party (M.Y.H.).

### Study Selection

The criteria for inclusion in the meta-analysis were (1) studies investigating patients undergoing intravascular ultrasound (IVUS), (2) reporting coronary WSS and plaque morphology, (3) reporting types (severity) of WSS, (4) over 6 months completed follow-up period, and (5) enrolling human population. Exclusion criteria were (1) noncoronary WSS, (2) insufficient statistical data for effect size, (3) studies not in humans, (4) studies in children, and (5) articles not published in English. Biplane coronary angiography and virtual histology-IVUS were used to accurately show the artery in 3D and to measure blood flow. The 3D anatomy of the artery was reconstructed from digitized radiofrequency IVUS signals and 2 planes of coronary angiography. The arterial lumen and outer vessel wall were reconstructed from digitized and segmented end-diastolic IVUS frames as previously described.^11^ Based on the reported WSS units expressed as dynes/cm^2^, different types were classified as: low (<10 dynes/cm^2^), intermediate (≥10-25dynes/cm^2^), and high WSS (>25 dynes/cm^2^).^12^

### Outcome End Points

Key clinical end points were the relationship between coronary plaque morphology and WSS. Main outcome measures were changes in coronary plaque features: lumen area, plaque area, necrotic core area, dense calcium area, fibrous area, and fibrofatty area, as well as assessing plaque vulnerability. The plaque was considered more vulnerable if it developed all 3 following features at follow-up: increased necrotic core area, decreased fibrous and fibrofatty area, and expansive remodeling.^13-15^ Based on the ACC Clinical Expert consensus documents on standards for acquisition, measurement, and reporting of IVUS studies,^12^ the different patterns of remodeling were classified as excessive expansive (profound), compensatory, and constrictive remodeling. Positive Delta external elastic membrane (EEM) area was defined as positive remodeling, and negative Delta EEM area was defined as constrictive (negative) remodeling. Furthermore, segments with positive remodeling were subdivided into excessive expansive (Delta EEM area divided by Delta plaque area, ie, plaque area at follow-up minus plaque area at baseline was >1) or compensatory (Delta EEM area divided by Delta plaque area was between 0 and 1.0). Constrictive or negative remodeling is also called adaptive remodeling, and it is more commonly seen in atherosclerotic stable lesions unlike positive remodeling which is characterized by more unstable lesions. Compensatory (also called compensatory expansive or incomplete) and excessive expansive (profound) remodeling is called overcompensatory and suggests greater plaque vulnerability.

### Data Extraction

Eligible studies were reviewed and the following data were abstracted: (1) first author’s name, (2) year of publication, (3) study design, (4) severity of WSS (high WSS, intermediate WSS, and low WSS), (5) follow-up duration, (6) patients demographic characteristics, (7) age and gender of study participants, and (8) IVUS measurements including: lumen area, plaque area, necrotic core area, dense calcium area, fibrous area, and fibrofatty area, in different types of WSS.

### Quality Assessment

Assessment of risk of bias in the studies included in the analysis was evaluated by the same investigators for each study and was performed systematically using the Quality Assessment of Diagnostic Accuracy Studies questionnaire (QUADAS-2) optimized to our study questions (Supplementary file 1).^11^ The QUADAS-2 tool has 4 domains for risk of bias: patient selection, index test, reference test, and flow and timing, and 3 domains for applicability: patient selection, index, and reference test domains.

### Statistical Analysis

The meta-analysis was conducted applying the conventional statistical analysis models using the RevMan (Review Manager [RevMan] Version 5.1, The Cochrane Collaboration), and a 2-tailed *P* < .05 was considered significant. The number of patients, means, and standard deviations (SDs) were pooled to weighted mean difference (WMD) and a 95% CI. Baseline characteristics are reported in median and range. Mean and SD values were estimated using the method described by Hozo et al.^12^ Analysis is presented in forest plots, the standard way for illustrating the results of individual studies and meta-analysis. Meta-analyses were performed with a fixed-effects model, and a random effect was used if heterogeneity was encountered. Heterogeneity between studies was assessed using Cochrane Q test and *I*^2^ index, as a guide, *I*2 <25% indicated low, 25% to 50% moderate, and >50% high heterogeneity.^16^ To assess the additive (between-study) component of variance, the reduced maximum likelihood method (τ^2^) took into account the occurrence of residual heterogeneity.^17^ Publication bias was assessed using visual inspections of funnel plots and Egger test. For studies with differences in sample size, we used influence analysis to show whether any study significantly altered the direction of association between different types of WSS and plaque morphology.

## Results

### Search Results and Trial Flow

Of 2488 articles identified in the initial search, 549 studies were screened as potentially relevant. After excluding 524 studies on the basis of title/abstract as not relevant, unrelated to study objective, animal studies, review articles, letter to editor, or language other than English, the remaining 25 full-text articles were considered for inclusion in the meta-analysis. After careful assessment, 18 of the 25 articles were further excluded according to the eligibility criteria (Table 1) leaving 7 articles to be included in the final analysis^5,6,18–22^ (Supplementary file 2).

**Table 1.** Main Characteristics of Trials Included in the Study.

Study year	Study design	Types of WSS	No. of segments	Inclusion criteria	Exclusion criteria	Primary endpoints
Samady et al. 2011	Observational prospective study	Low WSS	2249	Abnormal noninvasive Stress test or angina syndrome	MI, cardiogenic shock, hemodynamic instability, CABG, PCI	Lumen/plaque
		Intermediate WSS				Necrotic/dense Ca
		High WSS				Fibrous/fibrofatty
Stone et al. 2012	Observational prospective study	Low WSS	1341	CAD in at least 1 coronary segment requiring PCI/1 vessel suitable for IVUS	Clinical unstable LM/multivessel disease coronary calcification preluding IVUS	Lumen/plaque
		Intermediate WSS				
		High WSS				

Corban et al. 2014	Prospective observational study	Low WSS	2249	Abnormal noninvasive stress test or stable angina syndrome	NR	Plaque area
		Intermediate WSS				Necrotic/dense Ca
		High WSS				Fibrous/fibrofatty
Timmins et al. 2015	Prospective observational study	Low WSS	3871	CAD	NR	Plaque area
		Intermediate WSS				Necrotic/dense Ca
		High WSS				Fibrous/fibrofatty
Hung et al. 2017	RCT	Low WSS	1843	CAD non-STEMI	Cardiogenic shock EF<30%, prior CABG significant VHD	Lumen/plaque
		Intermediate WSS				Necrotic/dense Ca
		High WSS				Fibrous/fibrofatty
Timmins et al. 2017	Observational prospective study	Low WSS	14235	Abnormal noninvasive stress test or angina syndrome	NR	Plaque area
		Intermediate WSS				Necrotic/dense Ca
		High WSS				Fibrous/fibrofatty
Kok et al. 2019	Observational prospective study	Low WSS	2488	Abnormal noninvasive stress test or stable angina syndrome	NR	Plaque area
		Intermediate WSS				Necrotic/dense Ca
		High WSS				Fibrous/fibrofatty

Abbreviations: Ca, calcium; CABG, coronary artery bypass graft; CAD, coronary artery disease; EF, ejection fraction; IVUS, intravascular ultrasound; LM, left main; MI, myocardial infraction; non-STEMI, non-ST elevation myocardial infarction; NR, nonreported; PCI, percutaneous coronary intervention; RCT, randomized controlled trial; VHD, valvular heart disease; WSS, wall shear stress.

#### Characteristics of included studies

Seven studies (1 randomized clinical trial [RCT] and 6 observational) with 615 patients and 28 276 arterial segment measurements and with median follow-up 7.71 months (6-12) were finally included in the analysis. The mean age of the included patients was 56.8 ± 8.9 years (66% male), 69% of whom had arterial hypertension and 26.5% had diabetes (Table 2).

**Table 2.** Main Characteristics of Patients Enrolled Among Trials Included in the Study.

Study year	Groups	No. of patients	No. of segments	Age year	Male %	HTN %	DM %	TC mg/dL	Triglyceride mg/dL	Smoking %
Samady et al. 2011	L-WSS	20*	205	54 ± 10*	65	70	35	186 ± 13	115.5+	25
	I-WSS		1034	NR	NR	NR	NR	NR	NR	NR
	H = WSS	27*	1010	NR	NR	NR	NR	NR	NR	NR
Stone et al. 2012	L-WSS	506*	1341*	65 ± 10*	79*	63.3*	35*	180 ± 23*	111 ± 15*	49*
	I-WSS									
	H = WSS									
Cobran et al. 2014	L-WSS	20*	3851*	52 ± 10*	38*	67*	17	154 ± 21	NR	50*
	I-WSS									
	H = WSS									
Timmins et al. 2015	L-WSS	5*	3871*	62.1 ± 7.6	65.5	72.2	23.6	NR	NR	NR
	I-WSS			61.7 ± 10.2	79.6	67.7	13.0	NR	NR	NR
	H = WSS			62.9 ± 10.3	74.1	74.1	16.7	NR	NR	NR
Hung et al. 2016	L-WSS	20*	1843*							
	I-WSS									
	H = WSS									
Timmins et al. 2017	L-WSS	20*	1785	54 ± 10*	65*	70*	35*	186 ± 16*	107 ± 101*	25*
	I-WSS		413							
	H = WSS		929							
Kok et al. 2019	L-WSS	20*	2388*	54 ± 12*	65*	70*	35*	186 ± 18*	115 ± 98.4*	NR
	I-WSS									
	H = WSS									

Abbreviations: DM, diabetes mellitus; H-WSS, high wall shear stress; HTN, hypertension; I-WSS, intermediate wall shear stress; L-WSS, low wall shear stress; NR, nonreported; TC, total cholesterol.

#### Characteristics of coronary plaques

##### The impact of high WSS on plaque features

**Figure 1.**
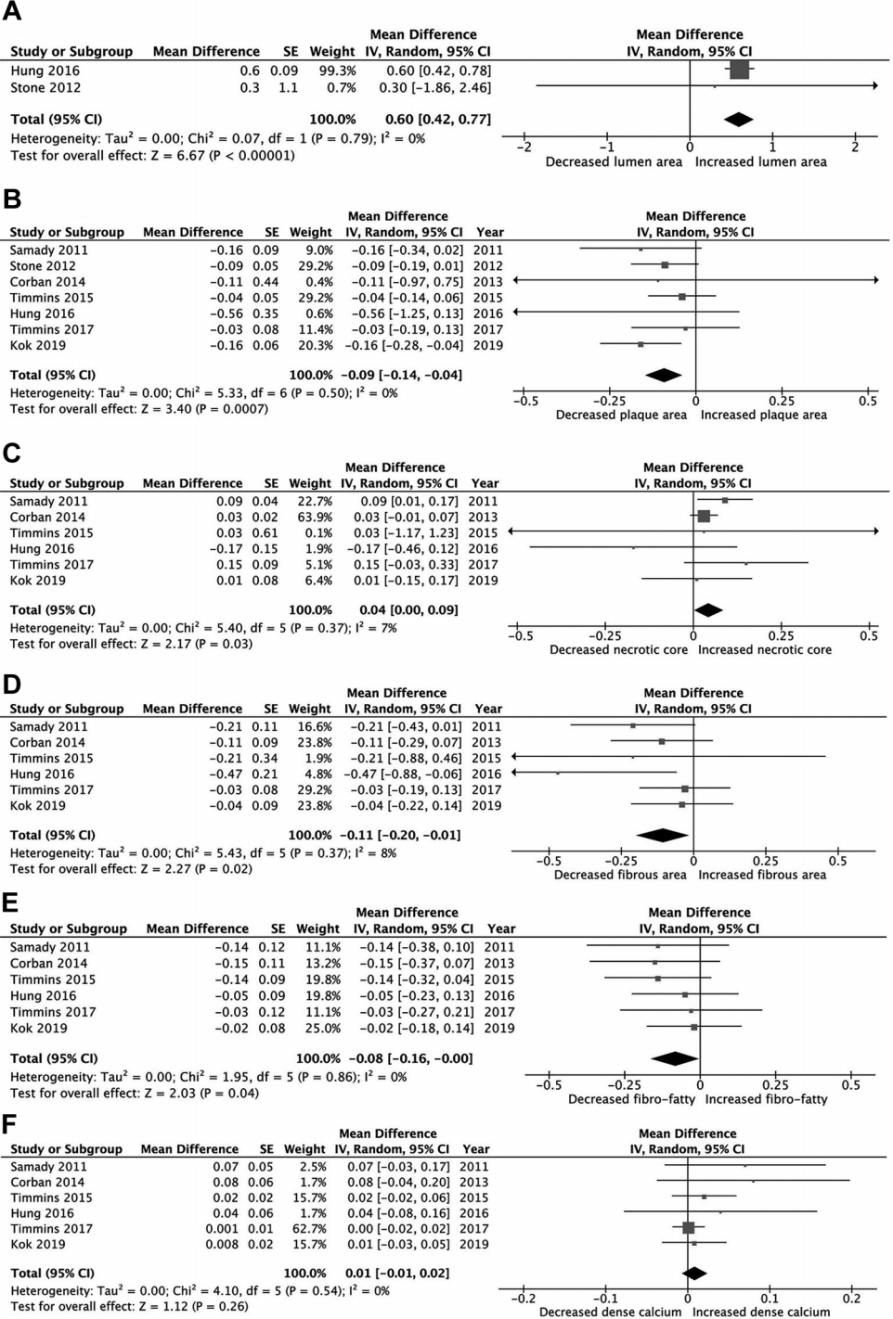
Mean changes in plaque morphology in the high wall shear stress (WSS) group: (A) lumen area; (B) plaque area; (C) necrotic core; (D) fibrous area; (E) fibrofatty area; and (F) dense calcium.

At follow-up, the pooled analysis showed high WSS to be associated with increased lumen area WMD 0.60 (95% CI: 0.42-0.72, *I*^2^ = 0.0%, *P* < .0001) and reduced plaque area WMD −0.09 (95% CI: −0.14 to −0.04, *I*^2^ = 0.0%, *P* = .007, Figure 1A-C). Specifically, high WSS was associated with regression of plaque fibrous area, WMD −0.11 (95% CI: −0.20 to −0.02, *I*^2^ = 8%, *P* = .02), fibrofatty area WMD −0.09 (95% CI: −0.17 to −0.01, *I*^2^ = 3%, *P* = .02), and increased necrotic core area, WMD 0.04 (95% CI: 0.01-0.09, *I*^2^ = 9%, *P* = .03), whereas dense calcium remained unchanged, WMD 0.01 (95% CI: −0.01 to 0.04, *I*^2^ = 0.0%, *P* = .026, Figure 1D-F).

##### Impact of high versus low WSS on plaque features

**Figure 2.**
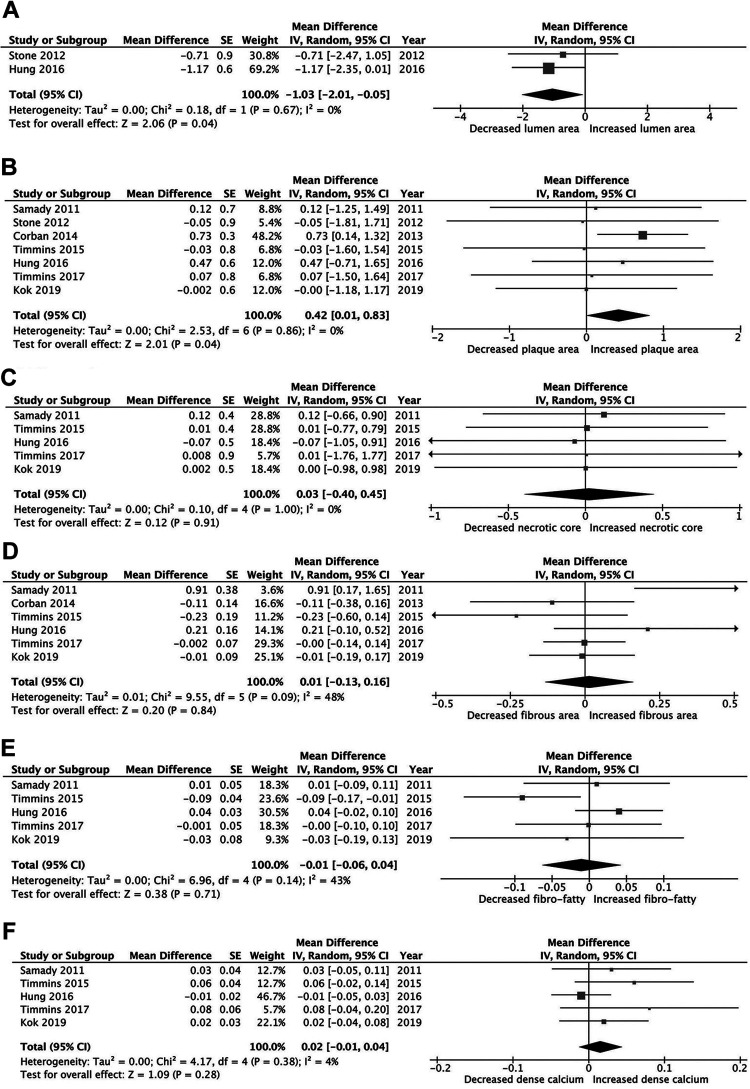
Mean changes in plaque morphology in the low wall shear stress (WSS) group: (A) lumen area; (B) plaque area; (C) necrotic core; (D) fibrous area; (E) fibrofatty area; and (F) dense calcium.

Compared with high WSS, low WSS showed different changes in plaque features. It was associated with decreased lumen area, WMD −1.03 (95% CI: −2.01 to −0.05, *I*^2^ = 0.0%, *P* = .03) and increased plaque area, WMD 0.42 (95% CI: 0.01-0.83, *I*^2^ = 0.0%, *P* = .04, Figure 2A-C). At follow-up, there was no regression in fibrous area, WMD 0.01 (95% CI: −0.13 to 0.16, *I*^2^ = 48%, *P* = .84), fibrofatty area, WMD −0.01 (95% CI: −0.07 to 0.05, *I*^2^ = 42%, *P* = .70), or necrotic core area, WMD 0.03 (95% CI: −0.40 to 0.45, *I*^2^ = 0.0%, *P* = .91). Dense calcium remained unchanged, WMD 0.02 (95% CI: −0.01 to 0.04, *I*^2^ = 4.0%, *P* = .28, Figure 2D-F).

##### Impact of intermediate WSS on changes in plaque features

Unlike low and high WSS, intermediate WSS was not associated with any change in arterial lumen, WMD −0.40 (95% CI: −1.29 to 0.49, *I*^2^ = 0.0%, *P* = .38), or plaque area, WMD −0.01 (95% CI: −0.15 to 0.16, *I*^2^ = 94%, *P* = .98, Supplementary file 3), fibrous area, WMD −0.01 (95% CI: −0.08 to 0.05, *I*^2^ = 0.0%, *P* = .64), fibrofatty area, WMD −0.40 (95% CI: −1.29 to 0.49, *I*^2^ = 0.0%, *P* = .38), necrotic core area, WMD −0.02 (95% CI: −0.03 to 0.07, *I*^2^ = 0.0%, *P* = .40), or dense calcium area, WMD −0.40 (95% CI: −1.29 to 0.49, *I*^2^ = 0.0%, *P* = .38 Supplementary file 4).

##### Features of plaque vulnerability according to the type of WSS

**Figure 3.**
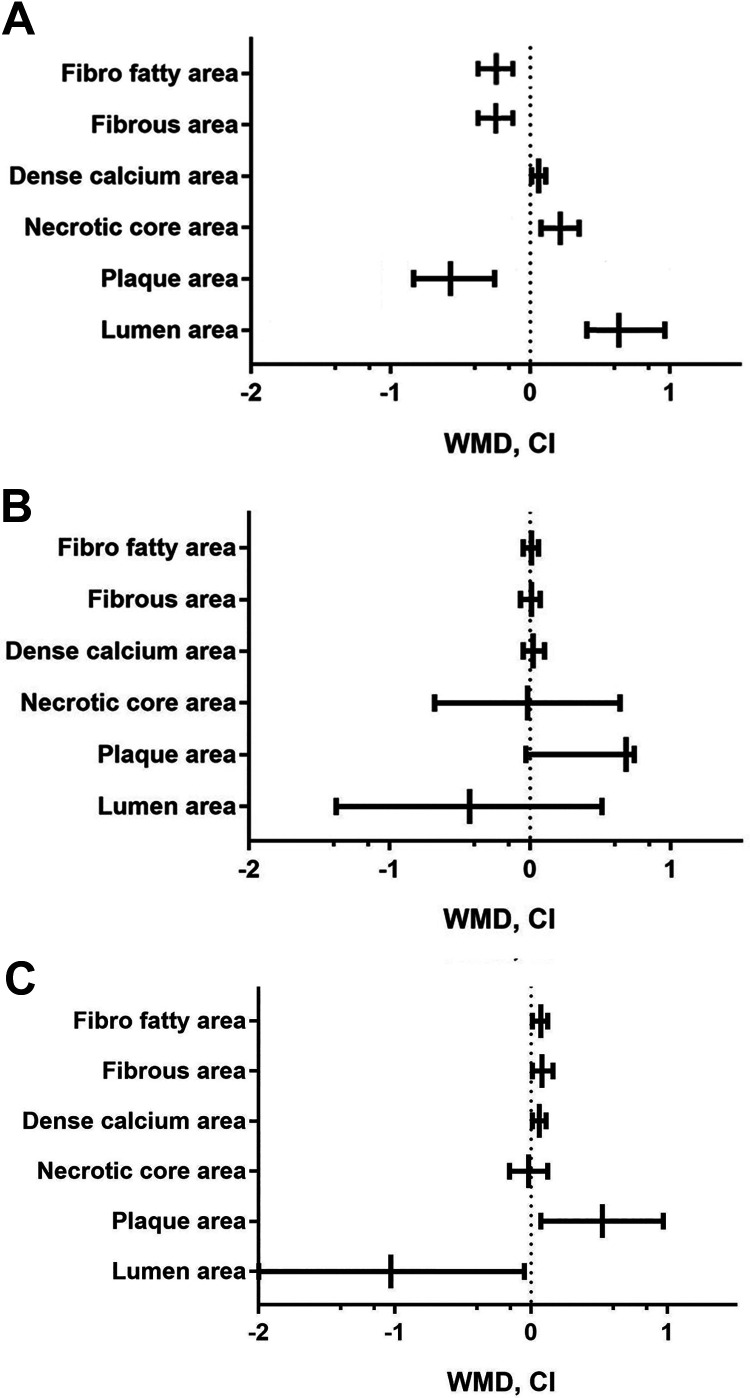
Mean change of plaque morphology in different groups of wall shear stress (WSS).

**Figure 4.**
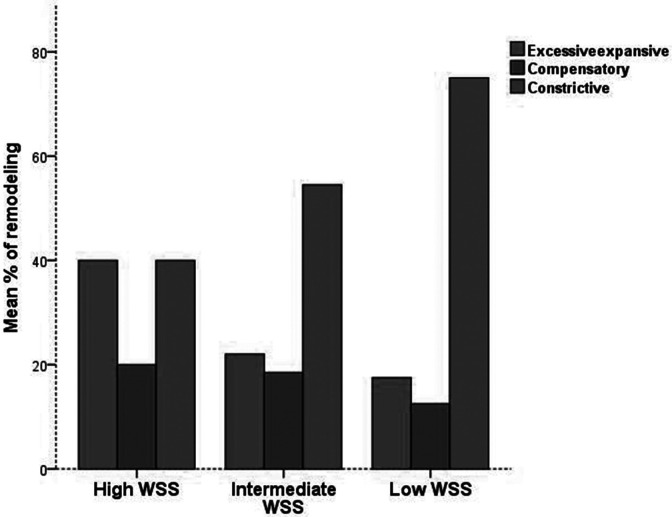
Arterial remodeling in different types of wall shear stress (WSS).

Compared with low WSS, high WSS was associated with clear features of worsening plaque vulnerability during follow up in the form of regression of fibrous (11% vs 1%) and fibrofatty area (10% vs 1%) and increased necrotic core area (5% vs 3%) Figure 3, Supplementary file 5. A high WSS resulted in development of more profound remodeling compared with low WSS (40% vs 18%, *P* < .05) which, in contrast, was associated with more constructive remodeling (78% vs 40%, *P* < .01, Figure 4). There was no significant difference in the vulnerability features of the plaques between low and intermediate WSS.

##### Risk assessment of bias

The assessment of risk of bias and applicability concerns based on the QUADAS-2 was optimized to our study questions (Supplementary 1).^13^ Four domains of criteria for risk of bias and 3 for applicability were analyzed, and the risk of bias was assessed as “low risk,” “high risk,” or “unclear risk.” Most studies had high or moderate level of quality and clearly defined the objectives and the main outcomes (Supplementary file 1, 6, and 7). Quality Assessment of Diagnostic Accuracy Studies questionnaire analysis for bias evaluation showed all domains to have low risk of bias (<30%), expected domain of applicability such as patient selection and index test that had high or unclear risk of 50%, due to lack of adequate exclusion and/or patient recruitment.

## Discussion

Earlier studies suggested that a high WSS may have a protective effect on endothelial function and described it as “normal wall shear stress.”^23-27^ In contrast, recent findings show a close relationship between high WSS and plaque vulnerability.^28-30^ Such controversial views require evaluation of the available evidence on the relationship between WSS and plaque features. This was our objective in this meta-analysis.

*Findings:* Our analysis of the available trials and studies showed that at least 6 months follow-up of high WSS resulted in significant changes in coronary plaque features and arterial lumen, with reduction in the plaque area and reciprocal widening of the arterial lumen. Intravascular ultrasound technique allowed identifying more detailed changes in plaque features that contributed to those gross arterial findings. High WSS was associated with regression of plaque fibrous area, fibrofatty area, and increased necrotic core area. Those changes described the profound arterial remodeling with high WSS. In contrast, none of such changes happened with low WSS despite its association with some constrictive remodeling. Of note, calcium density remained unchanged irrespective of WSS severity, high or low.

*Data interpretation:* Experimental studies have shown different remodeling response to low WSS,^30,31^ but prospective human studies demonstrated a consistent relationship between low coronary WSS and constrictive remodeling.^32-34^ Similar to those findings, our previous meta-analysis has shown that baseline high WSS is associated with clear features of vulnerable plaque such as higher necrotic core area and higher plaque burden compared to low WSS,^35^ which was related to constrictive arterial remodeling. Based on these findings, we designed the current study to further strengthen potential associations between different WSS and plaque feature changes over time in an attempt to establish a direct relationship. At the end of 6 months follow-up, our analysis showed significant worsening of features of plaque vulnerability only in patients with high WSS but not in those with low or intermediate WSS. This was clearly shown by increased necrotic core area and regression of fibrous and fibrofatty areas.^13-15^ These findings are supported by previous studies that showed high WSS as a contributor to plaque rupture in coronary and carotid arteries.^36-39^ Similarly, a randomized clinical trial with 3 years follow-up showed that high WSS could predict myocardial infarction in stable CAD.^30^ The effect of high WSS on the changes in plaque phenotype has been interpreted on the basis of stimulation of endothelial cell to produce transforming growth factor β that leads to apoptosis of smooth muscle cell and consequently thin cap and plaque vulnerability.^13,14^ Another impact is based on the increased nitric oxide production which leads to suppression of smooth muscle cells.^14^ In addition, shear stress has a mechanotransduction effect on the endothelium that involves several sequential steps: first, physical deformation of the cell surface; second, intracellular transmission of stress; third, conversion of mechanical force to chemical activity (“true” mechanotransduction); fourth, downstream biochemical signaling with feedback.^40^ The studies we analyzed did not have enough details to allow us to identify the most likely mechanisms responsible for increased plaque vulnerability with high WSS.^40-44^ In addition, high WSS causes more expansive remodeling compared with low WSS, thus implying more gross changes in plaque vulnerable features.

*Limitations:* The main limitation of this study is that there was only 1 RCT and a small sample overall. Another limitation is the lack of calcium classification measurements from spotty calcification to extensive calcification and any comparison with angiographic calcification and clinical outcome. Oscillatory WSS and its relationship with plaque features changes was not consistently reported in the included studies; therefore, it was not analyzed. The feasibility of proposing regular use of IVUS in routine clinical practice is a practical limitation.

*Clinical implications:* Our findings support the important role of low WSS in maintaining stable coronary arterial wall function and remodeling. Although IVUS is a costly investigation for measuring WSS, computed tomography coronary angiography future software development may be an alternative for routine applications.

## Conclusions

High WSS in the coronary circulation is associated with worsening features of plaque vulnerability and the development of more profound arterial wall remodeling compared with low WSS.

## Supplemental Material

Supplemental Material, sj-pdf-1-ang-10.1177_0003319721991722 - High Coronary Wall Shear Stress Worsens Plaque Vulnerability: A Systematic Review and Meta-Analysis Click here for additional data file. Supplemental Material, sj-pdf-1-ang-10.1177_0003319721991722 for High Coronary Wall Shear Stress Worsens Plaque Vulnerability: A Systematic Review and Meta-Analysis by Artan Bajraktari, Ibadete Bytyçi and Michael Y. Henein in Angiology
