# Supplementary material for: High Coronary Wall Shear Stress Worsens Plaque Vulnerability: A Systematic Review and Meta-Analysis
Source: Angiology. 2021 Feb 4;72(8):706–14. doi: 10.1177/0003319721991722 (PMC8326896; doi:10.1177/0003319721991722)

Supplementary data

Supplementary 1. Qualitative assessment of study reporting

Domain	Questions	Judgements
**Risk of bias**		
1) Patient selection	*Was a consecutive or random sample of patients enrolled?*	Yes, No, Unclear
	Was a case-control design avoided?	Yes, No, Unclear
	Did the study avoid inappropriate exclusions?	Yes, No, Unclear
	Could the selection of patients have introduced bias?	Low, High, Unclear
**Applicability** 1) Patient selection	*Is there concern that the included patients do not match the review questions?*	Low, High, Unclear
**Risk of bias** 2) Index test	*Were the index test results interpreted without knowledge of the results of the reference standard?*	Yes, No, Unclear
	If a threshold was used, was it pre-specified?	Yes, No, Unclear
	Could the conduct or interpretation of the index test have introduced bias?	Low, High, Unclear
**Applicability** 2) Index test	*Is there concern that the index test, its conduct, or interpretation differ from the review question?*	Low, High, Unclear
**Risk of bias** 3) Reference standard	*Is the reference standard likely to correctly classify the target condition?*	Yes, No, Unclear
	Were the reference standard results interpreted?	Yes, No, Unclear
	without knowledge of the results of the index test?	Yes, No, Unclear
	Could the reference standard, its conduct, or its interpretation have introduced bias?	Low, High, Unclear
**Applicability** 3) Reference standard	*Is there concern that the target condition as defined by the reference standard does not match the review question?*	Low, High, Unclear
**Risk of bias** 4) Flow and timing	*Was there an appropriate interval between index test(s) and reference standard?*	Yes, No, Unclear
	Did all patients receive a reference standard?	Yes, No, Unclear
	Did patients receive the same reference standard?	Yes, No, Unclear
	Were all patients included in the analysis?	Yes, No, Unclear
	Could the patient flow have introduced bias?	Low, High, Unclear

Patients: Patients with CAD; different types of WSS.
Index test: Relationship between types of WSS and plaque morphology
Comparator test (if applicable): Features of vulnerability of coronary artery in different types of WSS during follow up.
Target condition: Role of types of WSS in coronary plaque; Reference standard: morphology of coronary plaque

WSS: Wall shear stress; CAD: Coronary artery disease

Supplementary 2. Flow chart of study section

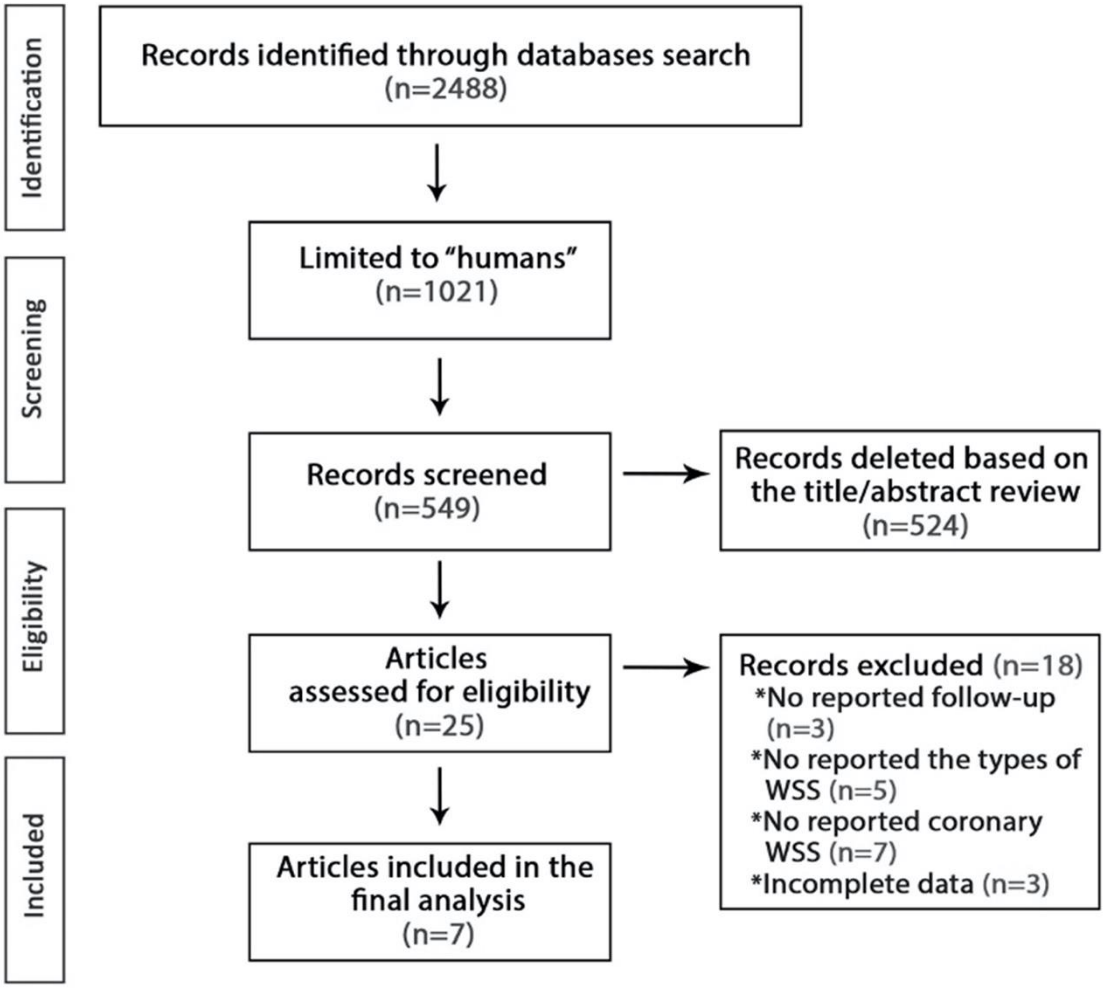

Supplementary 3. Mean changes in plaque morphology in the intermediate WSS group: a) lumen area; b) plaque area; c) necrotic core.

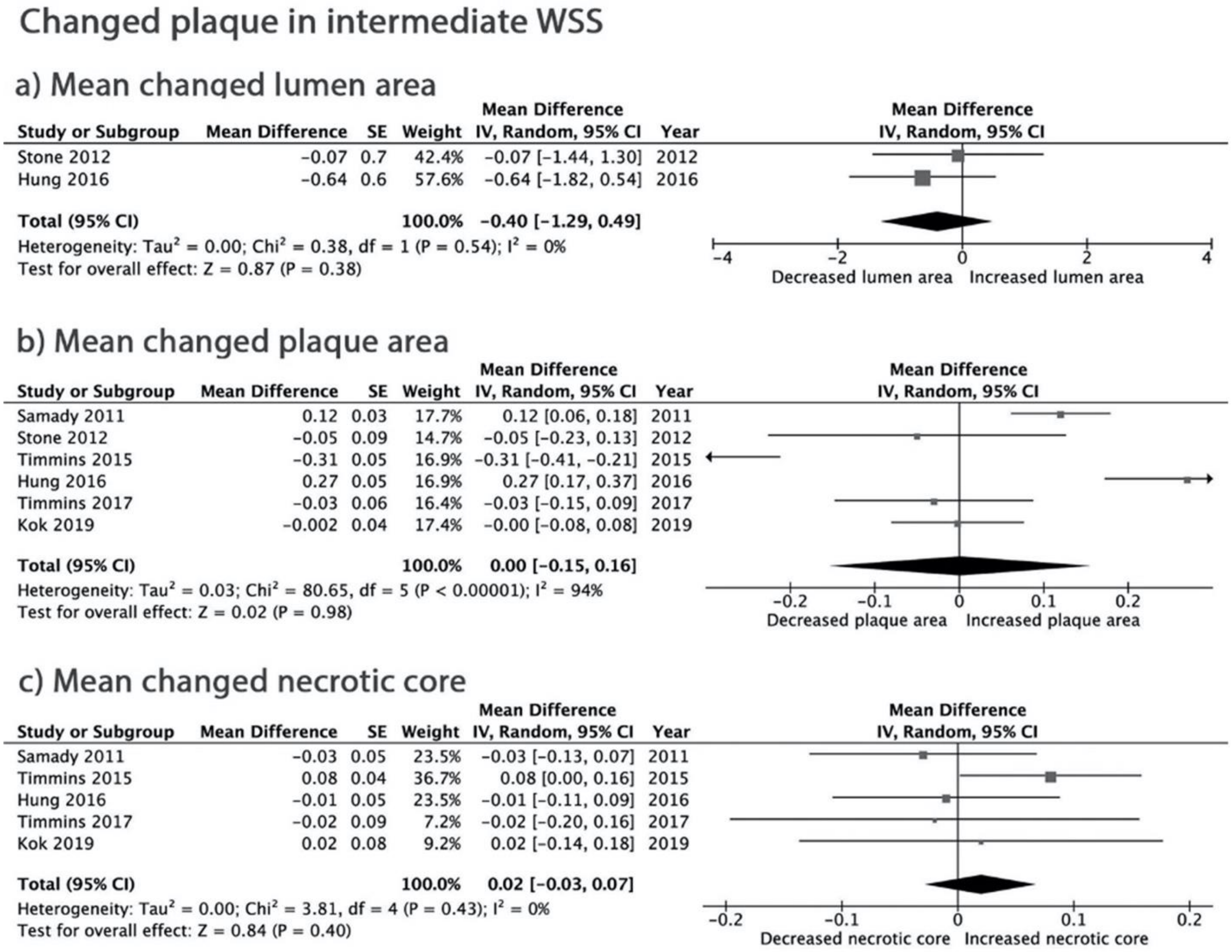

Changed plaque in intermediate WSS

a) Mean changed lumen area

Study or Subgroup	Mean Difference	SE	Weight	Mean Difference IV, Random, 95% CI	Year
Stone 2012	−0.07	0.7	42.4%	−0.07 [−1.44, 1.30]	2012
Hung 2016	−0.64	0.6	57.6%	−0.64 [−1.82, 0.54]	2016
**Total (95% CI)**			**100.0%**	**−0.40 [−1.29, 0.49]**	

Heterogeneity: $Tau^2 = 0.00$; $Chi^2 = 0.38$, df = 1 (P = 0.54); $I^2 = 0\%$
Test for overall effect: Z = 0.87 (P = 0.38)

b) Mean changed plaque area

Study or Subgroup	Mean Difference	SE	Weight	Mean Difference IV, Random, 95% CI	Year
Samady 2011	0.12	0.03	17.7%	0.12 [0.06, 0.18]	2011
Stone 2012	−0.05	0.09	14.7%	−0.05 [−0.23, 0.13]	2012
Timmins 2015	−0.31	0.05	16.9%	−0.31 [−0.41, −0.21]	2015
Hung 2016	0.27	0.05	16.9%	0.27 [0.17, 0.37]	2016
Timmins 2017	−0.03	0.06	16.4%	−0.03 [−0.15, 0.09]	2017
Kok 2019	−0.002	0.04	17.4%	−0.00 [−0.08, 0.08]	2019
**Total (95% CI)**			**100.0%**	**0.00 [−0.15, 0.16]**	

Heterogeneity: $Tau^2 = 0.03$; $Chi^2 = 80.65$, df = 5 (P < 0.00001); $I^2 = 94\%$
Test for overall effect: Z = 0.02 (P = 0.98)

c) Mean changed necrotic core

Study or Subgroup	Mean Difference	SE	Weight	Mean Difference IV, Random, 95% CI	Year
Samady 2011	−0.03	0.05	23.5%	−0.03 [−0.13, 0.07]	2011
Timmins 2015	0.08	0.04	36.7%	0.08 [0.00, 0.16]	2015
Hung 2016	−0.01	0.05	23.5%	−0.01 [−0.11, 0.09]	2016
Timmins 2017	−0.02	0.09	7.2%	−0.02 [−0.20, 0.16]	2017
Kok 2019	0.02	0.08	9.2%	0.02 [−0.14, 0.18]	2019
**Total (95% CI)**			**100.0%**	**0.02 [−0.03, 0.07]**	

Heterogeneity: $Tau^2 = 0.00$; $Chi^2 = 3.81$, df = 4 (P = 0.43); $I^2 = 0\%$
Test for overall effect: Z = 0.84 (P = 0.40)

Supplementary 4. Mean changes in plaque morphology in intermediate WSS group: a) fibrous area; b) fibrofatty area; c) dense calcium.

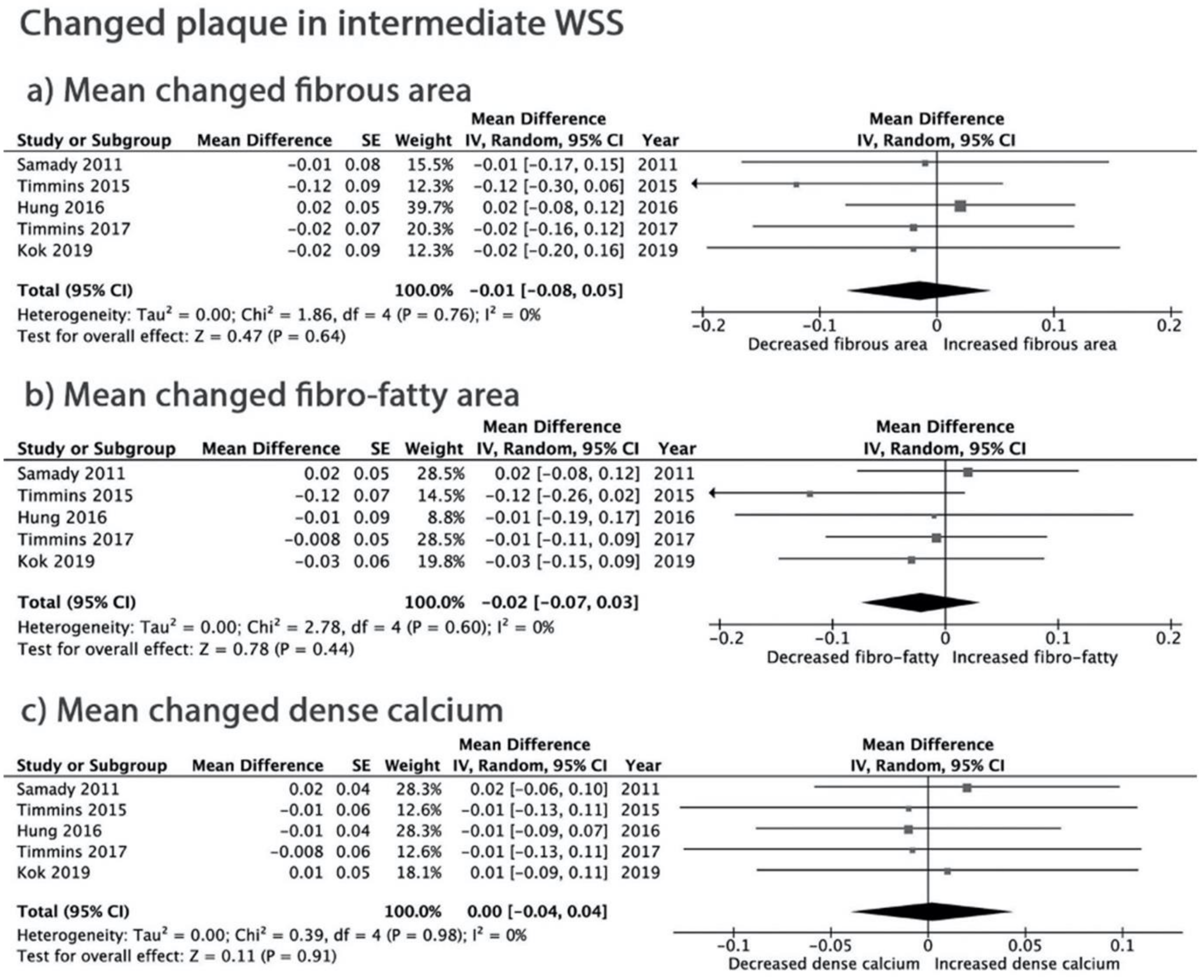

Supplementary 5. Role of wall shear stress in vulnerable plaque.

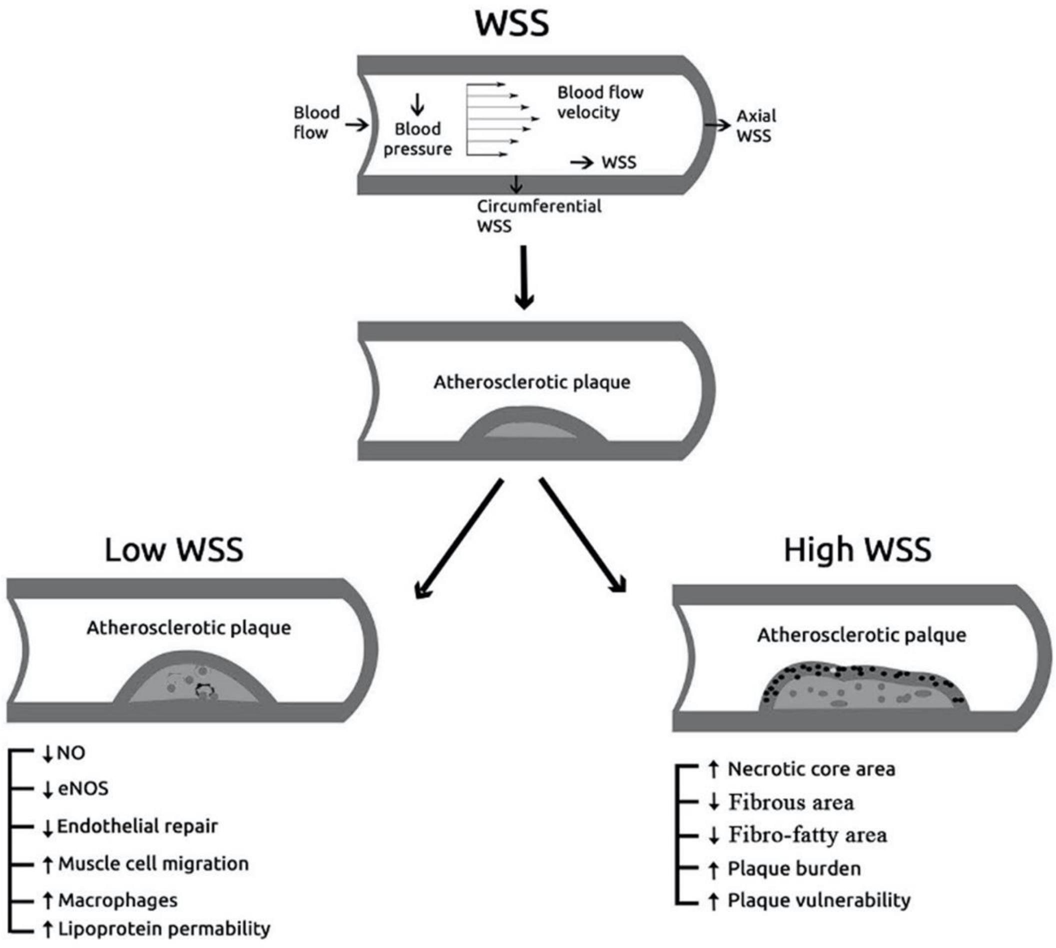

WSS: Wall shear stress; NO: nitric oxide; eNOS: endothelial nitric oxide synthase

Supplementary 6. Summary of QUADAS-2 Assessment of Selected Studies

**Author**	**Risk of bias**				**Applicability concerns**		
(year)	Patients selection	Index test	Reference standard	Flow and timing	Patients selection	Index test	Reference standard
Samady et al. 2011	Low	Low	Low	Low	Low	Low	Low
Stone et al. 2012	Low	Low	Unclear	Low	Low	Low	Unclear
Cobran et al. 2014	Unclear	Low	Low	Low	Unclear	Low	Low
Timmins et al. 2015	Low	Unclear	Low	Low	Low	Unclear	Low
Hung et al. 2016	Low	Unclear	Low	Low	Low	Low	Low
Timmins et al. 2017	Low	Low	Low	Low	Low	Low	Low
Kok et al. 2019	Low	Low	Low	Low	Low	Low	Low

QUADAS-2: Quality Assessment of Diagnostic Accuracy Studies-2.

Supplementary 7. Summary of quality assessment analysis (Quality Assessment of Diagnostic Accuracy Studies-QUADAS 2).

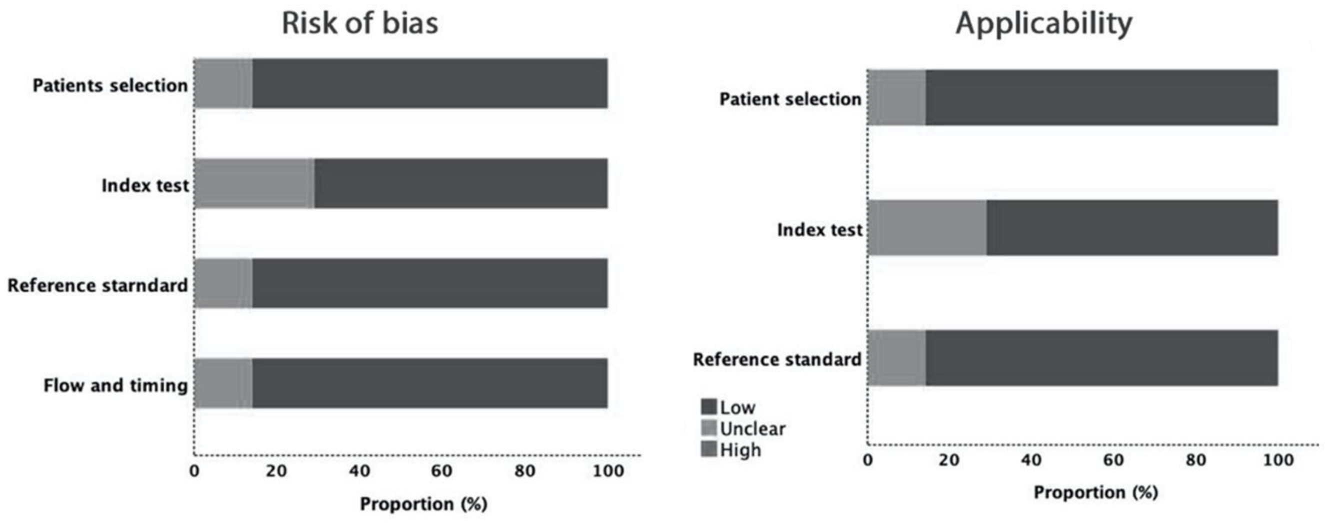

Supplement: Supplemental Material, sj-pdf-1-ang-10.1177_0003319721991722 - High Coronary Wall Shear Stress Worsens Plaque Vulnerability: A Systematic Review and Meta-Analysis [file sj-pdf-1-ang-10.1177_0003319721991722.pdf]
